# How prepared are healthcare professionals for delivering physical activity guidance to those with diabetes? A formative evaluation

**DOI:** 10.1186/s12913-019-4852-0

**Published:** 2020-01-03

**Authors:** N. Kime, A. Pringle, S. Zwolinsky, D. Vishnubala

**Affiliations:** 1 Academic Unit for Elderly Care and Rehabilitation, Bradford Institute for Health Research, Temple Bank House, Bradford Royal Infirmary, Bradford, BD9 6RJ UK; 2 College of Life and Natural Sciences, University of Derby, Derby, DE22 1GB UK; 3 West Yorkshire and Harrogate Cancer Alliance, White Rose House, West Parade, Wakefield, WF1 1LT UK; 4 NHS Vale of York Clinical Commissioning Group, GP Haxby Group, York, YO24 3BU UK

**Keywords:** Healthcare professionals, Physical activity, Diabetes

## Abstract

**Background:**

Physical activity is recognised as important for diabetes management and improved overall health of individuals with diabetes, yet many adults with diabetes are inactive. Healthcare professionals have been identified as key to promoting physical activity, including individuals with diabetes, but are ill-prepared to deliver this. Our paper evaluates the barriers/facilitators of healthcare professionals’ delivery of physical activity guidance to adults with diabetes and aims to inform efforts to investigate and enhance their preparedness to promote physical activity.

**Methods:**

A sequential mixed method, two-phase design was adopted involving a purposeful sample of healthcare professionals. Phase one was an online pilot survey designed to test assumptions around healthcare professionals’ knowledge, training and preparedness to deliver physical activity guidance. Phase two comprised eighteen semi-structured interviews, thematically analysed to provide an in-depth exploration of healthcare professionals’ experiences of delivering physical activity guidance to adults with diabetes.

**Results:**

Healthcare professionals are committed to promoting physical activity to adults with diabetes and are reasonably confident in giving basic, generic guidance. Yet, significant challenges prevent them from achieving this in their practice, including: lack of education and training around physical activity, diabetes and health; ignorance of recommended physical activity and diabetes guidelines; lack of awareness of referral options; limited time and accessibility to appropriate resources. Nevertheless, healthcare professionals believed discussions around physical activity needed to be an integral part of consultations, incorporating improved communication strategies for conveying key physical activity messages.

**Conclusions:**

HCPs have a key role in the promotion of physical activity to people with long-term conditions such as diabetes and they are identified within both the strategic policy context and national interventions for physical activity. Yet, this study indicated that HCPs face multiple and at times complex barriers to physical activity promotion generally and with diabetes patients. Conversely HCPs also reported what works, why and how, when promoting physical activity. Rich information derived from the day-to-day, working healthcare professional is integral to shaping future practices going forward. The bottom up, iterative design adopted in this study provides an approach to tap into this information.

## Background

Diabetes and its consequences are a major problem for health systems around the world, reaching epidemic proportions over the last 30 years [1]. Worldwide, over 420 million people are currently living with diabetes [2]. Furthermore, the absolute global economic burden of diabetes was $1.3 trillion in 2015 [3] and in 2016, around 1.6 million deaths were directly caused by diabetes [2]. Fortunately, diabetes and its consequences are - for the most part - manageable. Medication, regular screening, treatment for complications, improved physical activity (PA) levels and diet can all help to avoid, delay or manage diabetes [2]. Yet, much of the responsibility for facilitating the requisite behaviour change lies at the feet of healthcare professionals (HCPs) [4]. Although the evidence base surrounding theoretical frameworks and systems-based approaches to behaviour change point to the powerful impact of concurrently addressing underlying influences on behaviour [5–7], unhealthy practices are often socially reinforced [8, 9]. Therefore, effective, cost effective, sustainable and implementable strategies that can help alleviate pressure on HCPs and struggling health systems remain in short supply.

Contemporary national programmes for increasing PA focus on the promotion of PA, including for those people living with a long-term conditions, for example diabetes [10]. As part of national initiatives, such as Sport England’s Local Physical Activity Delivery Pilots, HCPs are expected to have an important role supporting both in the local community and community agencies tasked with promoting PA [10, 11].

While HCPs appear confident prescribing medication, screening, treatment and diet interventions for patients living with diabetes, there is less confidence and consensus among HCPs regarding the promotion of PA [12]. This could be problematic as the UK Chief Medical Officers (CMO) PA guidelines 2019 update, ‘developing options for future communication and surveillance’, highlighted the importance of a broad group of HCPs in promoting PA [13] and the Royal College of General Practitioners (RCGP) have identified PA as a strategic priority [14]. Many complex and multifaceted barriers account for insufficient levels of activity [15]. For example, HCPs do not always know what to recommend to people living with diabetes when asked about incorporating PA into their lives [16]. Further, HCPs and patients have cited this lack of knowledge, combined with the fear of hypoglycaemia and a loss of glycaemic control as a primary reason for non-engagement [17]. This fear may, in some way, account for people with diabetes being among the least physically active in society [18] and why a high proportion of patients living with diabetes struggle to maintain a healthy weight [19].

Allaying these fears are important since a physically active lifestyle is critical for blood glucose management and overall health in individuals with diabetes and prediabetes [20]. Adults with a high exercise capacity display reduced risk of coronary artery disease, myocardial ischaemia and stroke, regardless of their diabetes status [21]. In addition, compared to those individuals with type 1 who are inactive, their active counterparts have lower levels of retinopathy and microalbuminuria [22], an increased likelihood of reaching HbA1c, blood pressure and BMI targets [19] and decreased total daily insulin needs [23]. With this in mind and in line with current guidance, PA should be undertaken, recommended and prescribed to all individuals with a metabolic condition as part of diabetes management and overall health [24].

Adopting person-centred, behavioural approaches in this context may improve the chances of achieving the desired PA outcomes. These approaches indicate that regular positive experiences promote the motivation and capability to adopt a behaviour, whereas negative experiences can lower motivation and perceived capability [5, 25]. Highlighting the unique needs of adults with diabetes and the challenges they face around PA is likely to be of great importance. Previous research has highlighted the role that HCPs play in providing information and appraising social support in the form of education around diabetes and PA [15]. In addition, there is a growing need to improve knowledge around PA in relation to diabetes among HCPs [12, 16], alongside appropriate and individualised application of this, integrated within a programme that emphasises the importance of PA in diabetes management. Importantly, these ideas require a detailed understanding of the preparedness of HCPs for delivering guidance and support to participants, as well as a detailed appreciation of mediated approaches to PA promotion, developed for adults with diabetes. These approaches are expected to help to develop a better understanding of what works - or not, why and how. In doing so, bottom up, exploratory and iterative approaches have been recommended [26] and deployed with professional groups who have a responsibility for the provision of PA and public health [27]. This can help establish the basis for forming and refining intervention activities including those aimed at enhancing the preparedness of HCPs.

Therefore, the aim of the work was to conduct a formative evaluation of HCPs working in primary care and the community around the delivery of PA guidance for those with diabetes. The evaluation details information relating to the characteristics, decisions and behaviours of these HCPs, to inform and develop future education and training programmes for this group and, therefore, improve diabetes service provision in respect of PA. An important aspect of the study was to identify the challenges faced by HCPs and understand what worked less well and why, important criteria for the successful management of PA promotion interventions [28]. Here we describe this formative evaluation process and identify those factors that need to be considered prior to designing a behavioural intervention aimed at HCPs.

## Methods

### Study overview

This exploratory research study adopted a sequential mixed method design. The quantitative survey element of the research was implemented first and the findings were used to inform the qualitative interviews. As this study was exploratory, a non-probability sample of experts practicing within the field of diabetes and physical activity were recruited. This type of approach has been used previously in PA research to provide more in-depth accounts from health and social care professionals [27]. This involved a two phase, multi-site approach to evaluate the delivery of PA guidance by HCPs - working in either primary care or the community - to patients living with diabetes (including type 1 diabetes, type 2 diabetes and pre-diabetes). HCPs working in secondary care were not involved in this study. The study took place in the Yorkshire region between February and July 2018. A purposive sampling strategy was used to recruit HCPs who saw patients with diabetes as part of their role. Inclusion and exclusion criteria were based on whether a HCP was qualified, which was determined by the Health Care Personnel Law and Legal Definition. This states that Health Care Personnel are persons who have special education on health care and who are directly related to provision of health care services. The inclusion criteria were HCPs who met this definition.

Participants were first approached through General Practice surgeries and the researchers’ existing links with community organisations. An initial email was sent to HCPs inviting them to participate by the research team. Those that wanted to take part were given an information sheet and asked to provide written consent.

The first phase of this study utilised an online pilot survey with HCPs. It was designed to test assumptions around HCPs knowledge, levels of training and preparedness to deliver PA guidance to patients living with diabetes. The findings from this pilot phase were used to determine the most appropriate lines of questioning and issues to be unpicked during the semi-structured interviews in phase two. The second phase of the study was based on a phenomenological approach that focused on HCPs experiences [29]. This was the most appropriate theoretical framework for exploring HCPs understanding of PA and diabetes and their preparedness for delivering it to adults with diabetes. Phase 2 involved individual, semi-structured qualitative interviews with HCPs. The interviews provided an in-depth exploration, as recommended by Knight et al. [16], of HCPs experiences of delivering PA guidance to patients living with diabetes, giving them an opportunity to talk freely about their experiences. This combined approach facilitated a more robust and informative investigation of the current situation.

### Data collection

#### Online pilot survey

A small purposive sample of HCPs participated in the bespoke online pilot survey, hosted by Qualtrics [30]. The pilot included multiple choice and open text response questions on participants own engagement in PA, previous education, their work-based training and knowledge of PA and diabetes, and the practicalities of delivering PA guidance in an appointment setting. The pilot survey took approximately 15 min to complete.

#### Semi-structured interviews

Reflecting the importance of using iterative bottom up and exploratory approaches when understanding and investigating HCPs practices [26], individual semi-structured interviews were conducted face-to-face or by telephone. The research team facilitated the interviews, either individually or in pairs. Each interview lasted between 15 min and 1 h. They were arranged at a time to suit the participants and in the case of face-to-face interviews, were held either in the work place or the participants’ homes. An interview schedule was developed to guide the questions, which was piloted beforehand with a small number of HCPs. A semi-structured, deductive and inductive approach guided the interviews, which meant that the questions were aligned to both the main themes arising from the survey and the issues that arose directly from the participants as the interview progressed. This strategy allowed the participants to talk freely and share their views and experiences in respect of PA and diabetes. The questions centred on how prepared HCPs felt to deliver PA to those with diabetes, including the support they received and the barriers they faced. The interview schedule is available as supplementary material. Interviews were conducted until no new themes emerged and, therefore, data saturation was achieved. They were recorded with the participants’ consent and no participation incentives were offered. Appropriate University ethical and local research and governance approval were obtained.

### Data analysis

#### Online pilot survey

Descriptive statistics were used to describe data from the online pilot survey. All analyses were undertaken using IBM SPSS Statistics v25. Due to the sample size (*N* = 6) in phase one, there is an increased risk of disclosing information about individuals. For example, there are many cells with small counts, under 5. Although the tables themselves do not reveal the identity of an individual, there is a risk that combining or linking tables within this data set may result in a secondary disclosure. By adopting a policy of small number suppression, data cannot be reported from phase one. Nevertheless, data from the pilot study suggested that although many participants were physically active themselves, there were significant shortfalls in training and knowledge around PA promotion for people living with diabetes. As a result, confidence to deal with the practicalities of delivering PA guidance in an appointment setting appeared low.

#### Individual interviews

The interviews were transcribed, and qualitative data analysis was conducted using a thematic approach [31]. This process involved generating categories and coding data so that common themes and links could be identified, but at the same time ensuring the data remained a faithful representation of the participants’ comments [32]. As a means of reducing interpretation bias and increasing trustworthiness, transcripts were analysed by more than one member of the research team, all experienced in conducting qualitative analysis. In addition, participants verified the themes arising from the analytic process as a means of establishing the credibility of the findings. The researchers were aware of their influence on the study and endeavoured to employ reflexivity throughout the study process, using techniques such as critical reflection, note-taking and an assessment of their individual impact on data interpretation. A potential conflict of interest and ethical consideration was that one of the authors knew several of the participants, which may have led to coercive practices being employed. However, all participants were given an information sheet and asked to sign a consent form. The voluntary nature of participation was emphasised. In reality, the fact that some participants knew one of the authors turned out to be a strength of the study, since the author in question enabled access to a ‘hard-to-engage’ group, who ordinarily may not have set aside the time to participate in the study. Indeed, we came across cases where participants asked if they could be interviewed by this colleague and in doing so, we responded to participants’ needs and a trusting and safe relationship was established.

## Results

A total of 6 HCPs (4 Females; 2 males) participated in phase 1, some of whom participated in phase 2. A total of 18 HCPs (11 females; 7 males), participated in Phase 2. Of these, 15 participants were General Practitioners (GPs) and 3 were nurses employed either in a General Practice surgery on in the community. We present the results using a series of themes and selected excerpts in the following section which follows an approach we have used previously in the published literature [15].

### Individual interview results

#### Training/education for HCPs

One of the most striking aspects of the interviews was that all HCPs, except one, stated that they had received little or no pre-registration education in physical activity or exercise (PA/E), both as a mediator for a healthy lifestyle or for preventing and managing diabetes, as part of their undergraduate or post-graduate training. The little training that they had received around PA/E was generic, in terms of being “good for your health” rather than specific to diabetes. As one HCP commented,

“During medical training I received no physical activity training at all…So, if you then asked me specifically how much I should do or what I should be telling them [patients with diabetes], I’d probably just say, ‘you should do more’ and that would probably be about where my medical training ends on that front” (HCP 9).

The exception to this was one HCP, whose initial degree included information on PA/E and at-risk groups. Other than this, any training or education in PA/E and diabetes that HCPs had received was undertaken whilst in their current role as part of their continuous professional development (CPD). This was largely undertaken on an ad-hoc basis and by individuals who took the initiative and chose to complete self-directed learning, for example, e-learning modules on the British Medical Journal website or attendance at study days. However, unless HCPs had an interest in PA/E, it was not an area in which they would voluntarily seek training, as exemplified by these HCPs,

“It’s (PA/E) not in a GP curriculum, it’s not in GP training, it’s not in undergraduate training. So, unless you’ve found someone enthusiastic or somewhere to go and you’re interested in learning, it wouldn’t be something you’d do” (HCP 9).

“Well, I guess when it comes to diabetes specifically, we know a lot about the disease, the physiology, and I guess the drug management, but when it comes to the physical activity side of stuff I can’t think of a single time I ever had any kind of lecture or group session to do with that. I think physical activity in general is really poorly taught to doctors, and when I’ve had to learn stuff I’ve had to go and… It’s all very self-directed. I have actively to go and seek that information out. I can’t think of a single time either at medical school or during my… or my training when we covered those topics” (HCP 13).

For those HCPs who had completed CPD training on PA/E and diabetes, the focus was on type 2 diabetes, rather than type 1 diabetes or pre-diabetes. The emphasis was on medication and diet, and not PA/E, as a way of managing diabetes, as highlighted by one participant,

“There was never anything that was like here’s how to manage diabetes, on the lifestyle measure, physical activity. It was much more on the lines of, ‘right, diabetes, you need to discuss diet, you need to start thinking about medication’” (HCP 11).

Worryingly, even for those who had previously worked in a diabetes-specific role, PA/E was not a recognised component of diabetes management,

“I did actually do a diabetes job for four months. I wouldn’t say exercise was really discussed and since then, exercise-wise, pretty much zero CPD-wise” (HCP 8).

#### Guidelines for PA and diabetes care

All the participants were unfamiliar with any guidelines relating to PA/E and diabetes and had no idea where to go to find out specific information. Some participants referred to the British Medical Journal (BMJ) or the National Institute for Clinical Excellence (NICE), largely because they chose to be proactive, but the information sought mainly related to the treatment of diabetes rather than PA/E:

“There isn’t one consistent guidance for diabetes…I wouldn’t know if there was an authority to follow in the UK. NICE has a little bit of guidance about it, but again, it’s fairly broad” (HCP 9).

Of greater concern was the fact that only a small number of participants were familiar with the generic PA/E recommendations according to the CMO guidelines. Of these a few could recite the number of minutes for moderate and high intensity exercise per week, but as one HCP stated, this was despite having read any guidelines,

“I think I’d probably just have an image of what I’d say in my head, but that’s not based on being up-to-date…that I’ve read in the last 5 years” (HCP 10).

In addition, those HCPs involved in delivering medical training commented on the fact that even new doctors were unaware of the current PA/E guidelines for the general population,

“I was giving a talk to F1s (Foundation doctors) and I showed them the Government guidelines and the Chief Medical Officer guidelines [for PA/E]. We asked the question, ‘have you seen this before?’ I think one person put their hand up to say they’d seen it. It was a group of 30. No-one knew the guidelines” (HCP 9).

Given the lack of training and education or awareness of where to find information on PA/E and diabetes, some HCPs stated that they felt their day-to-day practice was compromised in respect of delivering PA/E guidance to their patients,

“I can’t speak for all doctors, but I have a feeling that the sentiment is shared, we kind of feel a bit out of our depth when it comes to physical activity in general, especially when it comes to physical activity and diabetes” (HCP 13).

Nevertheless, they provided advice to their patients, which was largely based on opinion rather than recommended guidelines or policy,

“I guess most of the guys I work with would say, ‘you should do more exercise’ or ‘you should eat better’. Everyone has an opinion, but it’s based on their opinions rather than necessarily formal training…so we give the advice that we think is best” (HCP 9).

#### Perceptions of practice for promoting PA with patients with diabetes

Even though most HCPs had not received any formal training in PA/E in general or as a means of managing diabetes, and furthermore, were unaware of current PA/E guidelines, HCPs believed that many of their peers were aware of the need for more people to adopt lifestyle recommendations. They referred to the importance of exercise, diet and losing weight as generic considerations for the population in general, but also, as a means of reducing the likelihood of developing pre-diabetes and type 2 diabetes. Some HCPs placed a greater significance on PA/E rather than medication as a more effective way of managing type 2 diabetes,

“I’m definitely a less is more doctor. If I can stop people’s medications, then I’m thrilled. But that’s why I sought it [PA/E] out, because there’s good evidence now that exercise and weight loss is probably at least as effective, if not more effective, than medication [for type 2]” (HCP 1).

A misconception as to what constitutes PA, as opposed to exercise, was prevalent amongst HCPs. When asked specifically about this issue, many believed there were a lot of mixed messages around PA, exercise and sedentary time, in terms of their meanings and recommendations for increasing activity. For those HCPs who could differentiate PA from exercise, they made a distinction between the two when advising patients and were able to emphasise the importance of fitting activity into everyday life,

“I also try to say they don’t need to specifically go out and exercise, but if they put some music on at home and they do lots of house jobs in that day, they actually use quite a lot of energy…Try and look at what you’re doing in a day and see where you can build in activity. When you do it regularly it becomes behaviour” (HCP 2).

Some HCPs believed that the existing PA/E support which was currently available was aimed at structured, formal exercise and not every day PA, when, in fact, the reverse should be the case with HCPs encouraging people to be more physically active within their everyday environment.

#### HCPs current practice

It was clear that there was no ‘one size fits all’ approach regarding HCPs delivery of PA/E guidance to their patients, irrespective of whether a patient had diabetes. Each HCP tackled PA/E differently depending on their role. Amongst GPs there were some commonalities. For example, they referred to the lack of time and not being able to fit PA/E, alongside other priorities, into one consultation. Therefore, the bulk of the responsibility for PA/E guidance was left to the practice nurses, although many GPs thought nurses probably placed a greater emphasis on diet rather than PA/E. When asked, GPs were unclear about the exact nature of the advice proffered by the practice nurses,

“I wouldn’t be 100% certain about how much time or information they’re [patients] given about exercise” (HCP 11).

In fact, practice and community nurses stated that they did address PA/E with all their patients who had diabetes, regardless of the type. This was achieved through an individualised, person-centred approach, focusing on a patient’s diabetes in the context of their lifestyle,

“I look at the person holistically – look at their medication, look at their current physical activity and look at the overall gains of physical activity to mental health, but also the weight loss and the impact that that will have on their physical health in reducing their blood sugars and the stabilisation of their diabetes” (HCP 6).

HCPs in general reported that of the patients they saw with diabetes, the majority had type 2 or pre-diabetes. Therefore, the emphasis was on weight loss or calorie counting through a combination of diet and PA/E. HCPs tried to give practical advice according to the individual and sought to ascertain: firstly, the level of PA/E that the individual was currently doing and secondly, determine whether the individual knew the recommended amount of PA/E that they should be doing. Finally, HCPs presented opportunities for being more active which were tailored to the individual’s lifestyle and, therefore, more likely to appeal to the individual,

“Then if we both thought they weren’t doing enough, or they’d like to do more to help their condition, I’d probably explore the type of things that they would be interested in doing, that they could sustain doing…rather than telling them what they should” (HCP 10).

#### Confidence in delivering PA/E advice to patients

Most HCPs felt reasonably confident in giving basic, generic PA/E advice to their patients. This consisted of exploring the activities that patients currently engaged in and encouraging patients to be more active in the context of their everyday lives. However, when asked about giving PA/E advice to those with diabetes, specifically type 2 diabetes, there was a divergence of opinion amongst HCPs. Some were reluctant to offer advice,

“On a 1-10 scale, with 10 being really confident, probably like 3-4. My advice to them would probably be really generic lifestyle and physical activity advice that I’d give to anyone. Specifically, how I’d tailor that to diabetes [type 2], I would not know how to” (HCP 13).

Others reported feeling confident that they could apply their knowledge, however limited, to pre-diabetes and type 2 diabetes, but unless they had received specific PA/E training in relation to type 1 diabetes, they lacked the confidence to deliver PA/E advice in this context,

“Someone who was pre-diabetic and overweight, I’d feel pretty confident. If there was someone who had poorly controlled type 1 who was on high doses of insulin, I’d feel quite nervous about giving too specific advice about how much physical activity they could safely do…I’d probably avoid the conversation altogether, to be honest” (HCP 10).

An important point raised by a few of the HCPs was the level of confidence that patients placed in their HCP. They felt that this was a significant factor in determining if a patient engaged in PA/E. Key to this was the perceived knowledge of HCPs around PA/E and the way information was delivered. Essentially, if HCPs seemed confident and knew what they were talking about, this instilled confidence in their patients,

“If you can get that engagement with them, that’s the other big thing. I think a lot to do with that is if they’re confident with you, you’re giving good messages…and they’re comfortable with you” (HCP 17).

#### Signposting and access to supportive resources

Most HCPs had limited knowledge about appropriate community services and support for patients who wanted to be more active. This was the case regardless of whether their patients had diabetes. They cited insufficient time to research what was available, an inability to keep track of local services and not knowing who to contact as the main factors,

“I wouldn’t actually know what’s available because it changes all the time according to funding and stuff in the area” (HCP 3).

Even when HCPs were aware of a local service, most notably, Exercise on Prescription, the National Diabetes Prevention Programme or DESMOND (Diabetes Education and Self-Management for Ongoing and Newly Diagnosed), they were unclear about how to access information to pass onto their patients,

“I found that there wasn’t very much information out there and that made it difficult for me to refer people in. People need to know…they’re asking you what it’s about and you think, ‘there’s only so much I can tell you’” (HCP 5).

In terms of in-house referrals, HCPs tended to signpost patients to either the practice nurse, visiting dietitian or in the case of one practice, a gym/personal trainer service.

The consensus was that if everything was in one location, there was an increased likelihood that patients would engage,

“I would argue that it (physical activity promotion) would come better from being from your GPs surgery than it would from the local authority. For the average lay person on the street, I suspect if they’ve got a health issue they’re not going to go on the council website, they’re going to come to us. And then if we send them to someone else to do it, that’s a barrier, in something that’s already got 20 barriers to doing the exercise” (HCP 1).

Some practices offered free education sessions for those with pre-diabetes or newly diagnosed type 2 patients. However, HCPs were unsure about the content of these sessions, whether PA/E was covered in the curriculum, and even whether they were effective,

“There’s no good scheme to refer them to. There’s a pre-diabetes education session, but it’s a one-off session and it’s theoretical. I don’t think it’s enough to make someone change” (HCP 9).

#### Future developments and improvements in PA and diabetes

All HCPs highlighted the need for a greater emphasis on PA/E, in medical school curriculums and as part of CPD, both generic and applied to specific conditions such as diabetes. In the patient consultation, HCPs thought that PA/E should be an integral component, rather than, at best, an add-on at the end. HCPs referred to the fact that currently, there is no requirement to discuss PA/E in a consultation because it is not linked to a target and, therefore, not incentivised,

“The only thing that drives me absolutely insane is the fact that the funding is completely the wrong way round. We’ve got QOF (quality and outcomes framework) targets for their HbA1c, their blood pressure, whether they’ve had their feet checked…and there’s no mention of exercise or weight loss. I would love the funding to be attached to sustained weight loss or sustained activity, or at least providing them with discussions about their activity. It would actually focus the problem on what’s actually underlying, rather than how they’re actually fixing it” (HCP 1).

When asked what HCPs needed to help them focus on PA/E in the consultation, they referred to concise information, i.e. a leaflet like the existing diet sheets, which was regarded as more convenient than having to look on a website,

“I’d probably say a one-page summary outline with reference to all the kind of key facts, with evidence supporting it, as to what we ought to be doing as clinicians for patients with diabetes, exercise and how to go about delivering that” (HCP 13).

Likewise, regarding patient education and PA/E, HCPs thought that information needed to be succinct with clear guidelines for the patient to follow. Also, tailored patient information was important. It was suggested that one way of achieving this was through an interactive patient hub or one-stop shop,

“Some sort of resource in that way in which you can click on to things and it takes you to something a bit more specific for you and gives you more tailored advice, would be useful” (HCP 18).

Many HCPs stated that PA/E promotion should be part of the practice nurse role rather than GPs since practice nurses saw patients with diabetes on a more regular basis. In addition, HCPs felt they needed improved strategies for communicating PA/E guidance effectively,

“Just getting the information across. May be some tips on how to get that information across and how to approach it with those patients, because they’re not always the most receptive to the information we try to give them” (HCP 12).

From an HCP perspective, time was often a constraint on whether they delivered any PA/E advice. HCPs stated that if they had up-to-date and readily available information on current PA/E recommendations and local services, they would be more likely to discuss PA/E in what was often a time-pressured consultation,

“For me it’s how we can get experts to make sure the resources are up-to-date, in an easy to find place, that’s marketed to the clinician and realistic to what we can do in practice” (HCP 9).

## Discussion

HCPs represent an important ingredient in efforts to promote PA within the population [13, 31, 33], including those with long-term conditions and this is reflected by their prominence in key national policy and interventions [10–14]. In this respect, understanding the barriers and facilitators that HCPs face are important in developing effective and supportive strategies and interventions that enhance their preparedness for PA promotion.

With those thoughts in mind, this study identified both the strengths of HCPs and the challenges they face in their efforts to promote PA for patients with diabetes. In line with previous research [34], our study encountered a genuine commitment by HCPs to promote PA despite difficult and challenging circumstances impacting their day to day work such as time [35, 36], large caseloads and competing demands as reported in other studies [37]. It has also been reported that medical students and doctors who are physically active themselves are much more likely to counsel their patients on PA and increasing the activity levels of medics has been suggested as an ingredient of a possible strategy for PA promotion in patients [38]. In this study, a number of HCPs reported in the interview data that they were physically active themselves and so this commitment to encouraging PA in their patients may also reflect their personal interest in sport and PA.

The commitment to PA promotion, including to those patients with diabetes is important, given the pivotal role of HCPs in national strategies and programmes designed for promoting PA, including those focused on patients with long-term conditions [10, 13, 39]. Indeed, the RCGP [14] identify PA as a strategic priority, while the 2019 CMO Physical Activity Guidelines have identified HCPs as being important in implementing PA messages to a range of groups [13]. Going forward, for the first time, the UK PA guidelines will be accompanied by a planned and coordinated communication strategy to support the implementation of the revised PA guidelines. Communication has been notably absent in recent PA guidelines and was recommended in technical reports aimed at supporting the new guidelines [40]. This is a positive development given HCPs in this study expressed concern about the fragmentation of resources to support PA advocacy.

That said, it is important that HCPs feel efficacious and knowledgeable in promoting PA to patients, including possessing an understanding of patients’ needs, motives and determinants, in addition to the current PA guidelines [34].

In the UK, multiple HCPs present with diverse levels of training and experience [41]. In this study we encountered instances where HCPs lacked knowledge on the recommended guidelines on PA, including PA and diabetes, as reported elsewhere [39, 42]. In these circumstances HCPs expressed understandable discomfort. A lack of knowledge around the PA guidelines has been identified for other conditions within the literature [43, 44], but also for diabetes. Indeed, Knight et al., [16] showed two thirds of HCPs were unfamiliar with the evidence-based guidance leaving them unable to offer basic advice on insulin action [41]. Furthermore, Cuthill and Shaw [38] have identified that clinicians’ knowledge of the relevant UK recommendations was reported in several studies to be as low as 7–27%. In part, this reflects the level of training and support HCPs receive, including training on PA which is an important component of effective PA promotion [39]. However, only very exceptionally did we encounter HCPs who had received full undergraduate training in PA and its relationship with health. Indeed a lack of training on PA promotion has been reported as a challenge, with HCPs in this study highlighting a lack of knowledge and skills as a result of little or no reference to PA in their undergraduate curriculum, which is documented elsewhere [38, 39, 45–47]. Interestingly enough, financial incentives have been suggested as interventions to incite HCPs to promote PA [39], yet we did not encounter this response in our study.

Looking forward, research has identified that future medical students want to receive more training on PA [48] and reports indicate that this issue is now receiving greater attention in undergraduate medical school curricula [49], for example, through Exercise Works! (http://www.exercise-works.org/). Yet this does little to solve the immediate training needs of HCPs currently in post, including those in our study, where other options are needed. Also, it is important to mention that having accurate knowledge of the dose of activity recommended for health benefits is not enough to translate into improved PA promotion for the population alone. Doctors and indeed all HCPs must be confident and competent in administering advocacy, promotion and counselling skills [38] and this is an important part of CPD strategies.

HCPs in this study reported that there were options to engage in CPD and indeed several participants identified professional bodies that offered CPD opportunities with accreditation and as part of nationally recognised programmes. This is a step in the right direction, but HCPs reported needing to be motivated to seek out these CPD opportunities, some of which they accessed through their own volition. With those thoughts in mind, the RCGP are collaborating with Public Health England on the GP Clinical Champion Programme for PA [50, 51]. This involves recruiting HCPs such as GPs, nurses and other allied HCPs to a ‘championing’ and ‘advocacy’ role for PA, where the incumbents establish and build new local networks to promote the case for PA promotion with fellow HCPs through training and education opportunities [52].

Given the importance of competence and confidence in PA advocacy [38], for those HCPs who expressed a lack of confidence in their ability to disseminate information and advice, the Clinical Champion Programme is a positive development, but a more developmental approach, such as mentoring or peer-led training, might also be valuable in refining the skills and competencies for PA advocacy. For example, a Sport and Exercise Medicine pilot is underway at Oxfordshire University Hospital Trust. The programme adopts a peer-led approach to training HCPs in PA across a variety of clinical pathways and an Active Hospital toolkit is also in development (https://www.sportengland.org/our-work/health-and-inactivity/moving-healthcare-professionals/).

A further component of PA promotion by HCPs is an awareness of PA opportunities. In this study, HCPs referred to a lack of awareness of local services and options for referring patients into local PA programmes operating in the community [53]. This is arguably exacerbated by major reform of public health both locally and nationally which in turn has implications for PA promotion. Nationally there has been a reduction in funding for local authorities for public health operation. The UK Comprehensive Spending Review reports that spending will fall by at least £600 million in real terms by 2020/21, an annual reduction of 3.9% [54, 55], resulting in funding for PA opportunities being scaled back in some areas [56]. The current economic climate may mean that the presence of PA opportunities such as Exercise Referral Schemes (ERS), which for a long time have been an important and readily available instrument in the PA armoury [38, 53] will be deemed resource intensive and may no longer be available. Already, this has meant that in some instances the commissioning process has led to a scaling back or restructuring of ERS in some local areas. Indeed, in one of the local contexts where this study took place, the local ERS had been restructured [57]. In some cases, this has reduced the number and type of options open for HCPs to refer patients to PA services. Changes in local PA provision may place further demands on HCPs to seek out the information on PA for patients from non-centralised sources, in turn contributing to time pressures faced by HCPs in their daily work and reported elsewhere [34]. Indeed, doctors report feeling insufficiently equipped to provide support or information to their patients [39]. In this study, online web-based sources of information on PA support and services were sometimes referred to as an easier option in providing information to patients, but knowing where to seek information was not always easy in practice, with information on PA located in multiple locations placing further time demands on already busy HCPs to seek out these resources. It remains to be seen if the updated 2019 PA guidelines [13], which is not only by a communication strategy, but also by a series of infographics available in centralised locations, helps address this challenge and would be a worthy focus of future investigations.

To support HCPs attempts to promote PA and in response to the pressures faced by HCPs, Moving Medicine (MM) was launched in 2018. MM is an interactive, evidence-based, internet tool to support brief advice in PA across a range of diseases and conditions [58]. Importantly, it houses information on PA in a central location. The intervention includes a series of modules linked to a range of conditions (including type 2 diabetes) in which PA can have a positive impact. It offers HCPs several strategies: time bound consultations, promotional materials and resources to assist patients through the process of being more physically active, as well as resources for patients that can be distributed. This is another step in the right direction and evaluation of this programme. However, given the responses of HCPs in this study, the evaluation should not only include impact outcomes on PA levels, but also process outcomes on the use and usability of resources [26, 59]. In particular, the extent to which MM helps HCPs overcome some of the commonly reported barriers in this study, such as confidence, competence time, knowledge and accessibility. Formative evaluative approaches like the one deployed in this study are likely to be valuable in this respect.

### Limitations and strengths

This study has several limitations and strengths. Limitations include a lack of representation from nurses who take on an important role in PA promotion [52, 60] and form an essential component in the dissemination of the updated UK CMO PA guidelines [13]. Many of the participants in this study thought PA was important. Efforts to promote PA for long term conditions are likely to involve a critical mass of HCPs including advocates and non-advocates. In that respect, it would have been insightful to have engaged those HCPs who felt PA was not important to better understand their reasons for this. This is also important in developing interventions that facilitate awareness, engagement and preparedness for PA promotion.

In thinking about how such activities are deployed, the authors encountered reluctance from some GPs to speak with outsiders or researchers from beyond their own professional circles. The strengths included an approach which identified a lack of presumed knowledge of the guidelines around PA and diabetes and an understandable anxiety amongst participants about this. This in part may have contributed to some of the difficulties when recruiting HCPs for this study, especially GPs. In overcoming this challenge, a further strength was the training of co-author and GP as a researcher who was involved in data collection. This helped address some of the preconceived misgivings about speaking with outsiders [61] preferring to speak with researchers who were both known and trusted and where a previous relationship existed. Furthermore, given the time pressures that busy HCPs faced in participating in this study, when they would otherwise be doing important tasks such as writing up patient notes and arranging referrals, the team adopted a flexible and accommodating approach around their availability. Equally important, this study provides some valuable insights into how to conduct research with this group and in this context.

## Conclusions

This evaluation presents new insights into the preparedness of HCPs for delivering PA guidance to adults with diabetes and also valuable information for how to undertake research in this setting and these groups. Using a sequential mixed method, two-phase approach, we have identified the factors underpinning the decision-making processes and behaviours of HCPs, as well as the challenges they face, when promoting PA to the diabetes community. Importantly, we have provided an opportunity for HCPs to tell their story through insightful accounts and through a trusted source. Given the importance of PA within the strategic and policy context for PA, rich information derived from the day-to-day, working HCP is integral to shaping future practices going forward. With those thoughts in mind, we provide the following recommendations.

## Recommendations

- Undergraduate education focused on PA and health conditions, including type 1 diabetes, type 2 diabetes and pre-diabetes.
- CPD opportunities for PA training and diabetes.
- Mentoring of HCPs who are new to the role or who just lack confidence in their ability to disseminate advice on PA to those with diabetes.
- Accessible, central database of current local PA providers.
- Tailored resources (web-based and printed) for HCPs and patients with diabetes.
- Consistent, joined-up approach between primary, secondary and community services regarding PA promotion for those with diabetes.
- PA services / programmes tailored specifically to patients with diabetes that are affordable.
- Continued and on-going dialogue with HCPs about their needs when promoting PA.
- Adopting a qualitative approach in investigating the barriers and facilitators that HCP face include non-supporters when developing interventions that help facilitate their engagement and involvement.

## Data Availability

The data generated and analysed during the current study are not publicly available due to the risk of individual privacy being compromised, but are available from the corresponding author on reasonable request.

## Abbreviations

BMI: Body Mass Index
BMJ: British Medical Journal
CMO: Chief Medical Officers
CPD: Continuous Professional Development
DESMOND: Diabetes Education and Self-Management for Ongoing and Newly Diagnosed
ERS: Exercise Referral Schemes
GP(s): General Practitioner(s)
HCPs: Healthcare Professionals
MM: Moving Medicine
NICE: National Institute for Clinical Excellence
PA: Physical Activity
PA/E: Physical Activity or Exercise
RCGP: Royal College of General Practitioners
